# Leber's Hereditary Optic Neuropathy: A Report on Novel mtDNA Pathogenic Variants

**DOI:** 10.3389/fneur.2021.657317

**Published:** 2021-06-09

**Authors:** Lorenzo Peverelli, Alessia Catania, Silvia Marchet, Paola Ciasca, Gabriella Cammarata, Lisa Melzi, Antonella Bellino, Roberto Fancellu, Eleonora Lamantea, Mariantonietta Capristo, Leonardo Caporali, Chiara La Morgia, Valerio Carelli, Daniele Ghezzi, Stefania Bianchi Marzoli, Costanza Lamperti

**Affiliations:** ^1^Unit of Medical Genetics and Neurogenetics, Fondazione IRCCS (Istituto di Ricovero e Cura a Carattere Scientifico) Istituto Neurologico Carlo Besta, Milan, Italy; ^2^Neuromuscular and Rare Disease Unit, Department of Neuroscience, Fondazione IRCCS (Istituto di Ricovero e Cura a Carattere Scientifico) Ca' Granda Ospedale Maggiore Policlinico, University of Milan, Milan, Italy; ^3^Neuro-Ophthalmology Service and Ocular Electrophysiology Laboratory, Department of Ophthalmology, Scientific Institute Auxologico Capitanio Hospital, Milan, Italy; ^4^Neuromuscular Disorders Unit, Fondazione IRCCS (Istituto di Ricovero e Cura a Carattere Scientifico) Istituto Neurologico Carlo Besta, Milan, Italy; ^5^Neurology Unit, IRCCS (Istituto di Ricovero e Cura a Carattere Scientifico) Ospedale Policlinico San Martino, Genoa, Italy; ^6^IRCCS (Istituto di Ricovero e Cura a Carattere Scientifico) Istituto delle Scienze Neurologiche di Bologna, Unità Operativa Complessa (UOC) Clinica Neurologica, Bologna, Italy; ^7^Department of Biomedical and Neuromotor Sciences, University of Bologna, Bologna, Italy; ^8^Department of Pathophysiology and Transplantation, University of Milan, Milan, Italy

**Keywords:** Leber optic atrophy, mitochondrial respiratory chain, complex I, LHON, transmitochondrial cybrids

## Abstract

Leber's hereditary optic neuropathy (LHON) is due to missense point mutations affecting mitochondrial DNA (mtDNA); 90% of cases harbor the m.3460G>A, m.11778G>A, and m.14484T>C primary mutations. Here, we report and discuss five families with patients affected by symptomatic LHON, in which we found five novel mtDNA variants. Remarkably, these mtDNA variants are located in complex I genes, though without strong deleterious effect on respiration in cellular models: this finding is likely linked to the tissue specificity of LHON. This study observes that in the case of a strong clinical suspicion of LHON, it is recommended to analyze the whole mtDNA sequence, since new rare mtDNA pathogenic variants causing LHON are increasingly identified.

## Introduction

Leber's hereditary optic neuropathy (LHON, OMIM #535000) is one of the most common inherited optic neuropathies causing bilateral loss of central vision. LHON is due to missense point mutations affecting mitochondrial DNA (mtDNA), usually found in the homoplasmic state, leading to mitochondrial dysfunction. Thus, LHON is maternally inherited but characterized by incomplete penetrance even in individuals carrying the same homoplasmic pathogenic mutation and with a clear male predilection (1).

Disease onset usually occurs during the second and third decades of life, but the age of onset can span from 2 to 87 (2). LHON typically presents as a painless, subacute, central vision loss in one eye, sequentially spreading to the other eye in weeks or months. Within 1 year, the large majority of affected patients have the second eye involved. Bilateral simultaneous onset occurs in about 25% of patients. During the acute phase, optic disc hyperemia, peripapillary-telangiectatic vessels, vascular tortuosity, and retinal nerve fiber layer (RNFL) pseudo-edema are often detected, even if these features may be subtle, with a slow progression toward blindness (3, 4).

Fundus changes can be accurately quantified by optical coherence tomography (OCT). In the acute phase, the RNFL initially thickens in the temporal and inferior quadrants and then in the superior and nasal quadrants (5). OCT shows a global RNFL thinning as disease progresses. Visual evoked potentials (VEPs) and pattern electroretinograms (PERG) are typically abnormal, as they reflect optic nerve fiber degeneration (6). The visual prognosis in affected patients is usually poor, also depending on the underlying pathogenic mutation, ranging from individuals declared legally blind, to others who experience spontaneous recovery of visual acuity (7).

Molecular diagnosis currently shows that about 90% of all LHON cases are due to one of three common mtDNA point mutations, at nucleotide positions m.3460, m.11778, and m.14484, defined as “primary mutations.” The diagnostic for other polymorphic variants for specific mtDNA backgrounds (haplogroup J) may be preferentially associated with some of the primary mutations (m.11778, and m.14484) exerting a synergistic modifying role; previously these variants were defined as “secondary mutations” (8). The most common is the m.11778G>A (*MT-ND4*, p.Arg340His) mutation, which accounts for ~70% of all cases, whereas the m.14484T>C (*MT-ND6*,p.Met64Val) and m.3460G>A (*MT-ND1*, p.Ala52Thr) mutations account for ~15% of cases (1). Recently, several rare or private mtDNA variants have been reported and validated, including the uncommon case of unique combinations of mtDNA polymorphic variants leading to a mild defect of complex I activity (9, 10). In view of these considerations, it has been proposed that, in case of evidence of maternal inheritance and clinical/OCT hallmarks of LHON, if none of the common primary mutations is found, the diagnostic gold standard should be the mtDNA complete sequence analysis (10).

In this report, we present eight patients affected by LHON belonging to five different and unrelated families of European descent, in which we identified five novel mtDNA variants, also providing some functional evidence of possible pathogenicity.

## Methods and Materials

Informed consent for a biological sample collection of fibroblast cell lines and DNA from blood to perform genetic studies was obtained from all patients involved, in agreement with the Declaration of Helsinki. The Ethical Committee of the Fondazione IRCCS Istituto Neurologico Carlo Besta, Milan, Italy, approved the study.

We have investigated five families with seven male individuals (patients 1, 3, 4, 5, 6, 7, and 8) and one female (patient 2) clinically affected by typical LHON but lacking any of the primary common mutations. A summary of the demographic, clinical, and genetic data is available in Table 1 (see the Supplementary Material for more detailed information).

**Table 1:** Schematic summary of patients'clinical, instrumental, and molecular examinations (see also Supplementary Material 1).

	**Family 1**		**Family 2**		**Family 3**		**Family 4**		**Patient 8**
	**Patient 1**	**Patient 2**	**Patient 3**	**Patient 4**	**Patient 5**	**Subject 1**	**Patient 6**	**Patient 7**	
Gender	M	F	M	M	M	F	M	M	M
Age range at onset	>40	>50	20–40	<20	20–40	20–40	20–40	20–40	>40
Affected eye	OU	LE	RE	UN	LE	NN	LE	OU	LE
Time to second eye involvement	NN	NN	Two months	UN	One month	NN	Three months	NN	One month
Clinical onset	Acute painless vision loss	Acute painless vision loss	Acute painless vision loss	Acute painless vision loss	Acute painless vision loss	NN	Acute painless vision loss	Acute painless vision loss	Acute painless vision loss
Alcoholic use Y/N	Y	N	N	UN	N	N	N	N	N
B12/folate bloodlevel	NR/NR	UN	NR/RD	NR/NR	NR/NR	NR/NR	NR/NR	NP	NR/NR
Smoke Y/N	Y	N	N	UN	Y	N	N	N	N
SD-OCT (average)	Normal PRNFL thickness OU (RE 108.25 LE 106.58 μm); Reduction of GCC thickness OU in perifoveal region (RE 84.43 LE 86.72 μm);	UN	PRNFL: RE focal increase of thickness in the superior pole (RE 120.06 μm); LE focal increase of thickness in the superior and inferior pole (LE 148.79 μm); diffused reduction of GCC in RE (63.12 μm), LE reduction in the perifoveal region (86.06 L μm)	UN	Reduction of PRNFL in OU In the inferior pole (RE 108.06 μm LE 92.50 μm); reduction of GCC thickness in OU (RE 86.63 μm; LE 92.62 μm)	NP	Reduction PRNFL in OU (RE 41.74 μm; L 42.54 μm); reduction of GCC thickness in OU in perifoveal region (RE 52.75 μm LE 56.99 μm)	Reduction of PRNFL in OU (RE 67.44μm; LE 69.89 μm); reduction of GCC thickness in OU (RE 58.32 μm; LE 54.01 μm);	Reduction of PRNFL in OU (RE 76.69 μm; LE 72.96 μm); reduction of GCC thickness in OU in perifoveal region (RE 71.32 μm; LE 71.25 μm)
VEP	OU: Increased latency and RD amplitude, more evident with pattern stimulus 15′ (papillo-macular bundle)	UN	Did not show a replicable response	UN	RE 60′ Irregular morphology and reduced amplitude, 15′ : not replicable response; LE 60′ e 15′ not replicable response	NP	RD amplitude and delayed P100 OU sx > dx (60′ e 15′)	RD amplitude and increased latency OU at 60′ and 15′	RE increase in latency and RD amplitude (60′); not replicable response (15′). LE not replicable response (60′ e 15′)
PERG	OU RD amplitude of N95	UN	OU RD amplitude of N95	UN	OU RD amplitude of N95	NP	OU amplitude RD of N95	RE not detectable; LE RD amplitude of N95	OU amplitude reduction of N95
Brain MRI	NR	NP	Enhancement of optic nerves bilaterally	NP	Bilateral hyperintensity of optic nerves, chiasm and part of the optical tracts.	NP	Unspecific, bilateral frontal white matter minimal alterations and bilateral thinning of optic nerves chiasm and optic tracts along with mild hyperintensity without enhancement.	Unspecific white matter lesions in T2-FLAIR infero-frontal lobe Left-side and anterior insular Right-side and iperT2 of the posterior part of the optic nerve without enhancement.	Mild swelling of the intraorbital ocular nerves portion.
Therapy (315 mg/die)	Idebenone (started 14 month after the exordium)	Idebenone (started 1 month after the onset)	Idebenone (started 5 month after the exordium)	NN	Idebenone (started 2 month after the exordium)	NN	Idebenone (started 8 month after the exordium)	Idebenone (started 12 years after the exordium)	Idebenone (started 4 month after the exordium)
Recovery	From finger count to 0.2 OU	NN	From finger count to 0.025 OU	NN	From 0.025 to 1 (RE) and 0.025 (LE) to 0.05 (RE) OS: 0.066 (LE)	NN	0.2 (RE), 0.1 (LE) without improvement	0.05 (RE) and 0.1 (LE) without improvement	From finger count (LE) and 0.6 (RE); to 0.025 (LE) and 0.025 (RE)
Mutation and localization	m. 13340 T>C, p.Phe335Ser on p.MT-ND5	m. 13340 T>C, p.Phe335Ser on p.MT-ND5	m.13379 A>G, p.His348Arg on p.MT-ND5	m.13379 A>G, p.His348Arg on p.MT-ND5	m.3632C>T, p.Ser109Phe on p.MT-ND1	m.3632C>T, p.Ser109Phe on p.MT-ND1	m.14538A>G, p.Phe46Leu on p.MT-ND6	m.14538A>G, p.Phe46Leu on p.MT-ND6	m.10350C>A, p.Leu98Met on p.MT-ND3

*BL, Bilateral; GCC, macular ganglion cell layer; F, female; LE, Left Eye; M, male; MRI, Magnetic Resonance Image; N, no; NN, none; NP, Not Performed; NR, Normal; OU, Both eyes; PERG, pattern electroretinogram; PRNFL, peripapillary retinal nerve fiber layer; RD, reduced; RE, Right Eye; SD-OCT, Spectral Domain Optical Coherence Tomography Analysis; UN, Undetermined; VEPs, Visual evoked potentials; Y, yes*.

Fibroblast cultures were obtained from skin biopsies of patients 3, 5, and 6 and from age-matched control subjects. Fibroblast cell lines were cultured in Dulbecco's modified Eagle's medium (DMEM) supplied with 10% fetal calf serum (FCS) at 37°C in a 5% CO_2_ atmosphere (11).

Trans-mitochondrial cybrids were generated from the fibroblasts of patient 6, as previously reported (12, 13).

Molecular analysis was performed on DNA extracted from peripheral blood lymphocytes of the eight patients and the unaffected individual. According to a standardized protocol (14) the entire mtDNA was PCR-amplified in eight overlapping fragments using a specific set of primer pairs. Each of the eight fragments was then sequenced, with four different “forward” primers, using a 3,100 ABI Prism Automated Sequencer. The mtDNA sequence was finally compared with the revised Cambridge reference mtDNA sequence.

Biochemical evaluation of OXPHOS complex activities was performed as previously described (14) in digitonin-treated skin fibroblasts from normal subjects and patients. The same measurements were performed in patient 6's cybrids and corresponding controls. The cells were cultured in a glucose-rich medium. The specific activity of each complex was normalized to citrate synthase activity, used as a standard mitochondrial mass marker.

The oxygen consumption rate (OCR) was measured in the fibroblasts of patients 3, 5, and 6 and in two controls using a SeaHorse FX-96 apparatus (Bioscience, Copenhagen, Denmark) as described in Invernizzi et al. (15) (data shown in Supplementary Figure 3). Cells were grown in a glucose-rich medium. Data are expressed as the mean ± SD, and comparisons were performed using a non-paired, two-tail Student *t*-test.

## Results

The complete sequence analysis of mtDNA from the eight subjects revealed five putative pathogenic LHON variants, one in each maternal lineage. All of them were absent in the mtDNA sequences from 100 healthy controls (screened and recorded in the in-house database from the Unit of Medical Genetics and Neurogenetics, The Foundation “Carlo Besta” Institute of Neurology). The m.13340 T>C (*MT-ND5*, p.Phe335Ser) variant was identified in patient 1 and in DNA from an affected first-grade female relative (patient 2) and the m.13379 A>G (*MT-ND5*, p.His348Arg) variant in patient 3 and his maternal relative (patient 4); both variants affect the ND5 subunit of complex I. Patient 5 and his asymptomatic first degree relative (subject 1) were shown to carry the m.3632C>T (*MT-ND1*, p.Ser109Phe) variant affecting the ND1 subunit of complex I. The relatives patient 6 and patient 7 carried the m.14538A>G (*MT-ND6*, p.Phe46Leu) variant in the ND6 subunit of complex I. Finally, we identified the m.10350C>A (*MT-ND3*,p.Leu98Met) variant in the ND3 subunit of complex I in patient 8. All variants were homoplasmic. The mtDNA haplogroup was determined for each family group using a specific provider (http//www.haplogrep.uibk.ac.at, not reported for privacy purposes, available on request).

All the identified variants affected evolutionarily highly conserved base pairs within the mitochondrial complex I subunits. More precisely, p.Phe335 and p.His348 are conserved in 97 and 94%, respectively, of more than 5,000 p.MT-ND5 sequences from protists to humans (16); from the same database, p.Ser109 results conserved in 80%, and p.Leu98 in 94% homolog sequences. However, p.Phe46 is only conserved in 16% of more than 5,000 p.MT-ND6 sequences. All the affected amino acid residues are located in transmembrane domains or adjacent to them. These variants are reported as probably damaging by most of the commonly used prediction software (Supplementary Table 1), although the predictions were not fully consistent; notably, the same results were observed even for other known LHON mutations.

**Figure 1:**
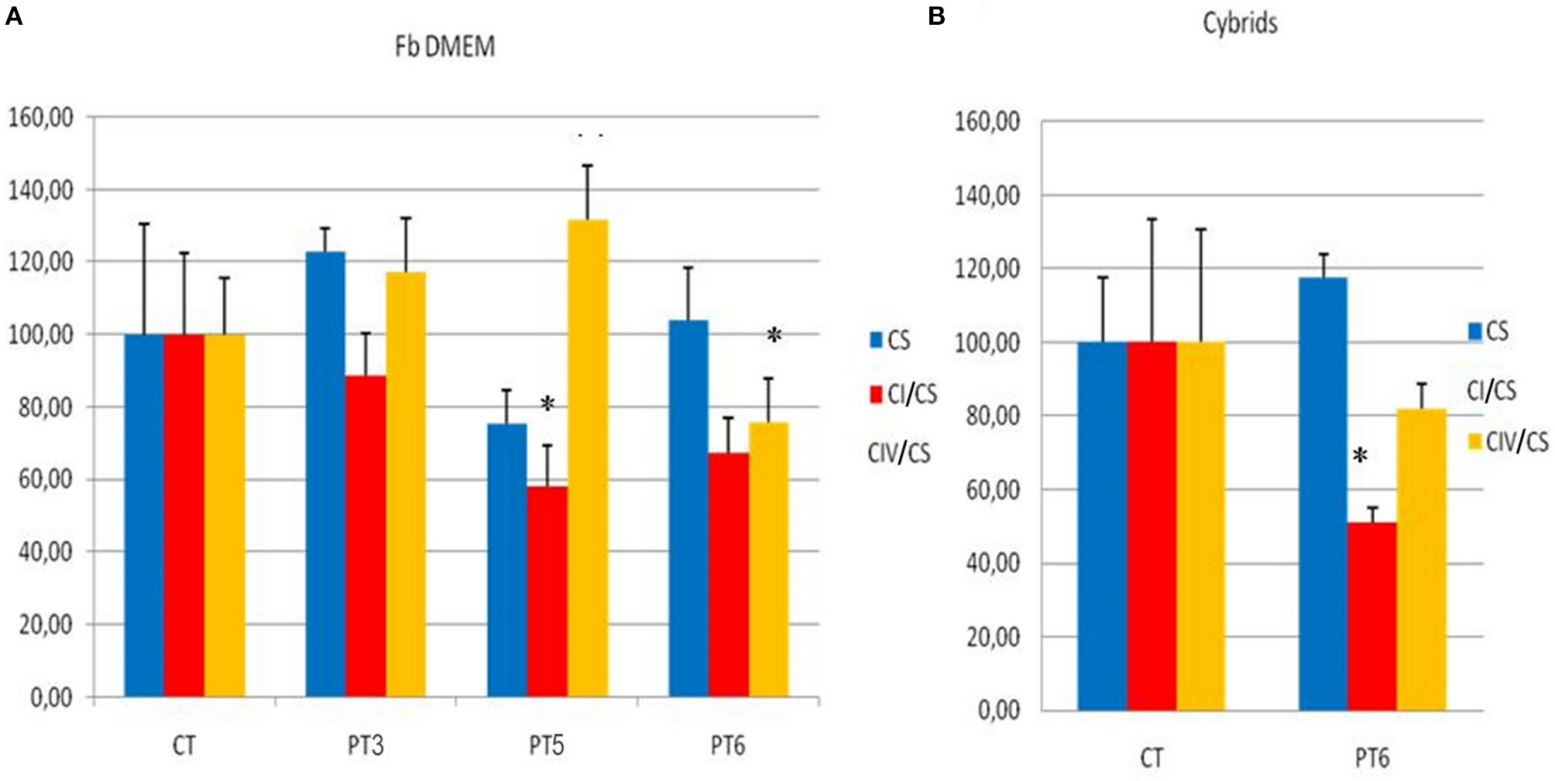
Mitochondrial respiratory chain activities on fibroblasts and cybrids. **(A)** Mitochondrial respiratory chain activities of CI and CIV complexes in Patient 3 (PT3), Patient 4 (PT4), Patient 6 (PT6) fibroblasts, and mediated controls (six different fibroblast cell lines) cultured in glucose-rich DMEM medium. **(B)** Mitochondrial respiratory chain activities of CI and CIV complexes in Patient 6 cybrids cultured in glucose-rich DMEM (PT6) and 5 mediated trans-mitochondrial cybrids controls (CT) grown in the same medium. Enzymatic activities (nmol/min/mg of protein) are expressed in percentage compared to controls and normalized with citrate syntase (CS) activity (blue column). CI, complex I (red column); CIV, complex IV (orange column). Student's *t*-test *p ≤ 0.05.

The mitochondrial respiratory chain activity in fibroblasts from three unrelated patients (patients 3, 5, and 6) harboring three different variants (p.His348Arg in *MT-ND5*, p.Ser109Phe in *MT-ND1*, and p.Phe46Leu in *MT-ND6*) showed a significant reduction in complex I activity in the fibroblasts from patients 5 and 6 (compared with age and sex-matched controls), whereas in patient 3 there was only a slight, nonsignificant reduction (Figure 1A). A statistically significant reduction of complex I activity was confirmed in cybrids of patient 6 compared with controls (Figure 1B).

Finally, the oxygen consumption rate (OCR) failed to show any clear difference in mitochondrial respiration rate in all patients analyzed (patients 3, 5, and 6) (see Supplementary Figure 3). SeaHorse respirometry was not performed on cybrids.

## Discussion

In this study, we report five putatively pathogenic mtDNA variants, all affecting ND subunits of complex I, in 8 patients form 5 unrelated families presenting with a classical LHON phenotype.

All of them are not present in diverse genomic databases (Mitomap, Ensembl, and Genome Browser) and were not present in the mtDNA from 100 healthy controls, suggesting a pathogenic association of these variants with LHON.

As further support to our hypotheses of pathogenicity, all these variants are extremely rare also in the set of 195,983 individuals from HelixMTdb (17) and 51,836 individuals from GenBank databases. In particular, m.3632C>T has been found only once in the heteroplasmic state in HelixMTdb and not once in GenBank databases; m.10350C>A has been found in the homoplasmic state only once in HelixMTdb and no individuals in Genbank; m.13340T>C has been found twice in the homoplasmic state and twice in the heteroplasmic state in HelixMTdb and in no individual from GenBank; m.13379A>G was not found in HelixMTdb nor in GenBank; finally, m.14538A>G was found once in the homoplasmic state in HelixMTdb and was not found in GenBank.

*In silico* prediction of pathogenicity using online resources classified the m.13340T>C, m.13379A>G, and m.3632C>T variants as functionally “deleterious”; the scores of pathogenicity were less consistent for the m.10350C>A variant and to a greater extent for the m.14538A>G variant. Four of them (m.13340 T>C, m.3632C>T, m.14538A>G, and m.10350C>A) have not previously been reported. Interestingly, the m.13379 A>G (*MT-ND5*) variant has been very recently found in an unrelated patient affected by typical LHON disease (18), thus representing an additional proof of its probable pathogenicity.

To further validate the pathogenicity of the mtDNA variants identified, we tested biochemical activity of respiratory complexes on both fibroblasts (from three patients) and cybrids (from one patient). We observed, to a variable extent, a reduction of complex I activity (Figure 1). SeaHorse respirometry, however, failed to show defective oxygen consumption. These results are not surprising; in fact, there is much evidence that even confirmed pathogenic LHON mutations may not display any detectable respiratory chain defect (19–22). High-resolution respirometry performed on fibroblasts from the recently described patient carrying the m.13379 A>G variant, gave ambiguous results, as well (15). The authors also report a high level of ROS production and a reduction of mitochondrial membrane potential (15).

It is worth considering that LHON is a tissue-specific disease, involving retinal ganglion cells and the optic nerve. Thus, the biochemical assessments performed on skin fibroblasts, a tissue not involved in the disease phenotype, may not necessarily mirror the respiratory activity of the tissue targeted by the disease. Besides, even for the three best-known primary mutations there is no validated biochemical method to prove their pathogenicity on fibroblasts.

The possible contribution of nuclear gene variants as molecular modifiers for the development of symptomatic LHON is not yet established and currently represents a challenging topic, worth further exploration. However, it has been previously reported that specific mitochondrial haplogroups, such as the haplogroup J, exert the role of penetrance modifiers in LHON (23). In fact, both LHON primary mutation m.11778G>A/*MT-ND4* and m.14484T>C/*MT-ND6*, when occurring on either a J1c or J2b mtDNA haplogroups, have increased risk of being symptomatic (23). In our cohort, one of the above-mentioned haplogroups was detected in patients 3 and 4. Notably, patient 3, despite Idebenone therapy, has been treated for more than 1 year but has not improved his visual function, supporting previous reports (23). Idebenone was administered to all affected patients (with exclusion of patient 4). All but patient 7, for whom therapy was started 12 years after the clinical onset due to misdiagnosis, received therapy roughly within the first year after onset. In five out of seven cases, idebenone treatment has been continued for more than 2 years with the exception of patient 2 (treated for slightly longer than 1 year) and patient 8, who has been treated for 5 months at the time of this study. We observed improvement in visual acuity in three out of six patients, stability in two, and a clear worsening in one (Table 1). In summary, therapy effectiveness for patients harboring these five novel mtDNA variants is in line with previously reported data on LHON due to “classic” mutations (1). Since the two patients in whom idebenone was ineffective carry the same m.14538A>G, we may speculate that this variant could be less responsive to therapy.

The peak age of disease onset was between the second and the third decades of life, and it is unusual for LHON patients to experience vision loss beyond 50 years of age, unless triggering factors have favored the disease onset as recently proposed (24). In relation to this observation, it is worth noticing that patients 1, 2, and 8 had late onset vision loss, the first around 50 and the third when he was almost 60. We may speculate that the variants in family 1 and patient 8 (m.13340 T>C and m.10350C>A) could be linked to phenotypes with later onset compared with classic LHON. Patient 1 was a heavy smoker and LHON vision loss occurred after at least 20 years of tobacco smoking and alcohol consumption (25, 26). However, familiar history supports pathogenicity of the identified variant, as a first-grade female relative (patient 2) affected by later onset LHON was found to be also positive for this variant. In patient 1 smoking and alcohol consumption could have contributed to accelerating the clinical onset. However, in patient 8, neither smoking nor alcohol abuse was reported; in this case, the identified variant (m.10350C>A) may be independently related to a later onset disease phenotype, or it could represent an additional predisposing factor within a specific genetic background or some other underrecognized triggering environmental/epigenetic influence. The latter is the most probable hypothesis, since we have an overall lower evidence of pathogenicity.

Possible variants on nuclear DNA have not been investigated in our group of patients because, with the exclusion of the recently reported *DNAJC30* gene (27), all the other nuclear genes have been associated with complex phenotypes rather than isolated Leber's hereditary optic neuropathy. *DNAJC30* has a “relevant” prevalence only in the Eastern European populations (none of our patients has such origins); moreover, it shows a recessive mode of inheritance that is not compatible with some of our pedigrees.

In conclusion, our study aims to emphasize the diagnostic relevance of running the whole mtDNA sequence analysis whenever LHON is documented on the clinical/OCT ground and the common mtDNA mutations are not detected, since rare or private mtDNA variants may be found (9). Besides, thanks to the advancement of the sequencing technologies, NGS is now widely available in most of the laboratories for mtDNA screening and is expected to become the method of choice for genetic analysis on mtDNA since it allows a rapid and cost-effective sequencing of the whole mtDNA with concurrent accurate quantification of heteroplasmy levels for point mutations (28). Remarkably, also, in our case series the novel mtDNA variants invariably affected ND subunits of complex I, and functional studies support a slight but detectable biochemical defect of complex I function in some of them. However, in all of them, we found relevant biological (low frequency of the identified variants in general population, evolutionary conservation, key location within complex I, and pathogenicity prediction) or clinical (typical course of disease and neuro-ophthalmological findings, family history) plausibility in support of their possible pathogenicity or potential contribution to pathogenicity in combination with other not yet determined genetic or extrinsic factors. Validation in further families will be necessary to consolidate the pathogenic role of these novel mtDNA variants in LHON. The possible role of other nuclear genes or environmental factors as disease modifiers needs to be further explored in future studies.

## Data Availability Statement

The original contributions presented in the study are publicly available. This data can be found here: https://zenodo.org/record/4683798#.YHfsV-gzaUk.

## Ethics Statement

The studies involving human participants were reviewed and approved by the Ethical Committee of the Fondazione IRCCS Istituto Neurologico Carlo Besta, Milan. The patients/participants provided their written informed consent to participate in this study.

## Author Contributions

LP and AC performed neurologic evaluations and wrote the paper. SM, EL, and MC performed *in vitro* studies. RF helped with patients selection. PC, GC, and LM performed all neuro-ophthalmological evaluations and contributed to manuscript preparation. AB, LC, and CLM assisted in data analysis and manuscript preparation. VC, DG, SB, and CL led the overall effort. All authors contributed to the article and approved the submitted version.

## Conflict of Interest

CL and VC are involved in LHON clinical trials with Santhera and GenSight Pharmaceuticals, serving also as consultants. The remaining authors declare that the research was conducted in the absence of any commercial or financial relationships that could be construed as a potential conflict of interest.

## Abbreviations

LHON: Leber hereditary optic neuropathy
RNFL: retinal nerve fiber layer
OCT: optical coherence tomography
VEPs: Visual evoked potentials
PERG: pattern electroretinogram
DMEM: Dulbecco's modified Eagle's medium
FCS: fetal calf serum
OCR: Oxygen Consumption Rate
CS: citrate synthase
SD-OCT: Spectral Domain OCT Analysis
ctrls: controls.
