# Supplementary material for: Leber's Hereditary Optic Neuropathy: A Report on Novel mtDNA Pathogenic Variants
Source: Front Neurol. 2021 Jun 9;12:657317. doi: 10.3389/fneur.2021.657317 (PMC8220086; doi:10.3389/fneur.2021.657317)
Supplement: Supplementary file 1 [file Table_1.docx]

Supplementary Material

1. **Supplementary data:** Patients’ detailed clinical information

Patient 1&2 (Family 1)

Patient 1 is a middle-aged Moroccan man and presented with a sudden onset of bilateral, painless, visual loss without any other sign. He had been a heavy smoker (20 cigarettes per day) and he overused alcoholic beverages for many years. Recollection of family history revealed that a first grade female relative (Patient 2) was also affected by the same disease. Parental consanguinity was not reported.

He underwent a complete neuro-ophthalmological examination six months after visual symptoms presentation that revealed a reduced bilateral visual acuity (Best Corrected Visual Acuity-BCVA bilaterally: count fingers); Ishihara testing showed a complete loss of color vision (score bilateral - BL: 0/16); HFA (Humphrey Field Analyzer) visual field testing was performed in both eyes. Testing of the RE (right eye) revealed a mean deviation score of -7.50 dB with central-scotoma; testing of the LE revealed a mean deviation score of – 7.68 dB with a centrocecal scotoma. Fundoscopy revealed a bilateral subtle edema of the optic nerve head with normal maculae.

OCT (Optical Coherence Tomography) analysis (RTVue 100) showed a normal thickness of RNFL (retinal nerve fiber layer) bilaterally (- RE 108.25; left eye- LE- 106.58 µm), with a BL (bilateral) thinning of GCC (ganglional cell layer) in perifoveal region (RE 84.43 LE 86.72 µm).

PERG (Pattern Electroretinography) were bilaterally normal. Brain MRI and optic pathways study were also normal. VEP (Visual Evoked Potential) showed increased latency and reduced amplitude, more evident with pattern stimulus at 15' (papillo-macular bundle), but normal P100 latency bilaterally. Blood tests were negative for hyperglycemia and B12/folate deficiency. Six months after the first evaluation he complained a worsening in visual acuity with impairment of daily-life activities. A new ophthalmologic examination showed a complete, bilateral, centrocecal-scotoma on automated visual field testing (HFA- 30-2) and color vision loss. OCT (RTVue 100) revealed a bilateral reduction of ganglion cell layer and macular thickness (Supplementary figure 1); VEPs showed increased latency and further reduced amplitude. PERG showed a BL amplitude reduction of N95. A new Fundus examination revealed a bilateral temporal pallor of both optic nerve heads associated with a reduction of RNFL thickness in R.

The three more frequent mutations associated with LHON (m.3460G>A, m.11778G>A and m.14484T>C) were excluded. The whole sequence of mtDNA showed a novel mutation in *MT-ND5* gene: m.13340T>C, leading to an amino acid change p.Phe335Ser, in a high conserved position. Patient received a therapy with Idebenone and stopped smoking and alcoholic use.

Idebenone therapy was started 14 months after the onset at high dosage (945mg /die) and the neuroophthalmological follow up revealed an incomplete bilateral improvement of visual acuity (BCVA BL: 0.2), no improvement in color vision testing and in visual filed testing was documented.

Patient 3&4 (Family 2)

A healthy, young adult caucasian man (patient 3) was referred to Neuro-Ophthalmological consultation because of sudden painless vision loss in the LE, following the occurrence of similar symptoms in the RE 2 month before. His medical history was unremarkable, without any parental consanguinity reported. He did not report trauma, medications or illicit drugs use nor smoking. Family history revealed that a maternal relative was affected by a not better specified optic neuropathy (patient 4). He underwent a complete neuro-ophthalmological examination that revealed a reduced bilateral visual acuity (BCVA BL: count fingers); complete loss of color vision on Ishihara testing (score BL: 0/16). HFA visual field testing was performed in both eyes. Testing of the RE revealed a mean deviation score of -28.28 dB with central-scotoma; testing of the LE revealed a mean deviation score of –24.57 dB with a centrocecal scotoma (Supplementary Figure 1). Intraocular pressure was normal. Fundus examinations revealed, in both eyes, a mild peripapillary nerve fiber layer pseudoedema and telangiectasia, more evident in the LE; the maculae and mid-peripheral retina were normal. MRI showed an abnormal T2 signal with mild enhancement along optic pathways bilaterally, in particular of chiasm and right optic tract. VEPs did not show any reproducible response. CSF examination was normal, aquaporin 4 antibodies were absent. Blood chemistry testing showed a mild reduction of serum folates. OCT (RTVue 100) revealed a focal increase of RNLF thickness in the superior pole of optic disc in RE (120.06 µm) and a more evident focal increase in superior and inferior pole of optic disc in LE (148.79µm); GCC was noticeably thinned in RE (63.12 µm) and in the perifoveal region in LE (86.06 L µm )-see table 1.

1 month after the onset of symptoms in the second eye, optic nerves became diffusely pale and peripapillary succulence switched to atrophy of nerve fibers layer. Patient 3 received coenzyme Q10, folin and Idebenone therapy. 18 months after theonset, visual function showed a slight bilateral improvement (BCVA BL: 0,025), no improvement in color vision testing and in visual filed testing.

The patient underwent a genetic screening for LHON and none of the common LHON mutations were found, but two novel homoplasmic mutations (m.8507 A>G and 13379A>G) were detected on the Atpase8 and *MT-ND5* genes, respectively. Therapy with Idebenone was started 5 months after the onset at high dosage (945mg /die) Follow up showed a bilateral improvement of visual acuity, (from count finger to 0.025).

The same genetic alteration was found in a maternal relative (now in his sixties). Unfortunately, neither clinical information nor instrumental examinations of patient 4 are available.

Patient 5&Subject1 (Family 3)

A young adult Caucasian man (Patient 5) presented with a sudden and painless visual loss in the LE, followed one month later by involvement of the RE. At that time the patient was a smoker (20 cigarettes per day). Upon hospital admission he underwent a CSF examination and aquaporin 4 antibodies test, both resulting in normal findings. Blood folic acid and B12 were normal.

He underwent a complete neuro-ophthalmological examination 4 months after the onset that revealed a reduced bilateral visual acuity (BCVA BL: 0.025); Ishihara testing revealed a complete loss of color vision (color vision BL: 0/16).

HFA visual field testing was performed in both eyes. Testing of the RE revealed a mean deviation score of -20.37 dB with centrocecal scotoma; testing of the LE revealed a mean deviation score of – 23.93 dB with central scotoma. Dilated fundus examination showed a mild bilateral temporal pallor of optic nerve heads with normal macula. OCT analysis (RTVue 100) revealed a bilateral thinning of RNFL in the inferior pole (RE 108.06 µm LE 92.50 µm) and a bilateral focal thinning of GCC, more evident in the temporal retinal region (RE 86.63µm; LE 92.62 µm).

VEPs showed an irregular morphology and reduced amplitude in RE at 60'; at 15' there was no reproducible response; VEPs were absent in LE. PERG showed BL reduced amplitude of N95.

Brain MRI showed a bilateral hyperintensity of optic nerves, chiasm with partial involvement of optical tracts.

Blood chemistry was normal., whole sequence of mtDNA was performed: a homoplasmic novel mutation in *MT-ND1* gene was identified (m.3632C>T), leading to an aminoacidic change p.Ser109Phe, in a highly conserved position.

He started Idebenone, 945 mg per day with mild recovery of visual function after 4 months.

The neuro-ophthalmological follow up revealed a bilateral improvement of visual acuity (BCVA RE: 0.1 LE: 00.66), no improvement was detected in color vision testing and in visual filed testing.

His asymptomatic first grade relative (Subject 1) was shown to harbor the same homoplasmic mutation in blood.

Patients 6&7 (Family 4)

A young adult Caucasian male (Patient 6) presented with an history of sudden, painless loss of vision in the RE at the age of 24, involving the LE after few weeks. Family history was negative for visual loss. Steroid therapy inefficacy ruled out an inflammatory hypothesis. Noteworthy, parental consanguinity was reported: the parents had the same surname and both were born in the same little village located in northern Italy.

He underwent a complete neuro-ophthalmological examination one year after the onset, that revealed a reduced bilateral visual acuity (BCVA R: 0.2, L: 0.1); Ishihara testing showed proper identification of 3/16 plates in R and 1/16 plates in L; HFA visual field testing was performed in both eyes. Testing of the RE revealed a mean deviation score of -17.00 dB with central-scotoma; testing of the LE revealed a mean deviation score of – 18.14 dB with a central scotoma.

Fundoscopy revealed a bilateral pallor of the optic nerve heads with normal macula and mild-peripheral retina.

OCT analysis (RTVue 100) revealed a bilateral thinning of RNFL (RE 41.74 µm; L 42.54 µm) and GCC in perifoveal region (RE 52.75µm, LE 56.99 µm)

VEPs showed reduced amplitude and delayed P100 bilaterally. Similarly, PERG amplitude was reduced bilaterally.

Brain MRI showed bilateral thinning of optic nerve chiasm and optic tracts along with mild hyperintensity without contrast enhancement. *OPA1* gene mutations were ruled out. NGS panel study analysis did not detect any pathogenic variants. Under the hypothesis of a LHON, we performed a whole mtDNA sequence, founding a novel homoplasmic variant (m.14538A>G, p.Phe46Leu) within *MT-ND6* gene.

A first grade relative (Patient 7) reported a similar clinical history, and carried the same homoplasmic variant. He underwent a complete neuro-ophthalmological examination, six months after the onset, that revealed a reduced bilateral visual acuity (BCVA R: 0.05, L: 0.1); impaired color vision (proper identification of 7/16 plates in R and 1/16 plates in L at Ishihara test); HFA visual field testing showed a mean deviation score of -19.23 dB in RE with central and superior scotoma and a mean deviation score of – 16 dB with a central and superior scotoma in the LE.

Fundoscopy revealed a bilateral pallor of the optic nerve heads, mild pallor of-peripheral retina with normal macular region.

OCT analysis (RTVue 100) revealed a bilateral thinning of RNFL (RE 67.44µm; LE 69.89 µm) and GCC (RE 58.32 µm; LE 54.01µm);

VEPs showed reduced amplitude and delayed P100 bilaterally. PERG were not detectable in RE, while amplitude of N95 was reduced in L

MRI brain showed iperT2-hyntensity of the posterior part of the optic nerve without contrast enhancement.

In both patients. Idebenone therapy was started (eight months and twelve years after the onset in patient 6, and patient 7, respectively), not leading to any detectable improvement after three years of treatment in both patients.

Patient 8 (family 5)

A Caucasian male in his late adulthood started to complain sudden and painless visual loss in his LE followed, after one month by painful visual loss in the other one. Family history revealed was positive for immunological disorders (Crohn, Addison disease, autoimmune thyroiditis and sarcoidosis) but not for ophthalmological diseases.

One month after the onset he underwent complete neuro-ophthalmological examination that showed a reduced bilateral visual acuity (BCVA RE: 0.6; LE: count fingers); impaired color vision in RE Ishihara testing score of 1/16 in RE, normal scores in LE).

HFA visual field testing was performed in both eyes. Testing of the RE revealed a mean deviation score of -9.15 dB with central and inferior scotoma; testing of the LE showed a mean deviation score of – 21.89 dB with central scotoma. Dilated fundus examination showed a mild temporal pallor of the optic disc in the RE. OCT analysis (RTVue 100) revealed a thinning of PRNFL in both eyes (RE 76.69µm; LE 72.96 µm) and of GCC (RE 71.32 µm; LE 71.25µm) in the perifoveal region.

RE VEPs showed an increase in latency and reduction in amplitude at 60' and no response at 15'. No reproducible response at 60' and 15' was registered in the LE.

PERG showed reduced amplitude of N95 bilaterally. Brain MRI showed a mild swelling of the intra-orbital tracts of ocular nerves. Neuro-ophthalmological follow up revealed a further deterioration of visual acuity in RE (BCVA: 0.025) and a mild improvement in LE (BCVA:0.025).

Color vision worsened in both (BL: 0/16). No improvement in HFA testing was detected. The patient was treated with steroid for 1 month without any improvement. After the onset of visual impairment in the right eye a new attempt with IV (intravenous) steroids was proven unsuccessful. CSF analysis and blood examination for aquaporin 4 antibodies resulted in normal findings.

mtDNA analysis excluded the three classic LHON mutations, but since a strong suspicion of LHON persisted, we performed whole mtDNA sequence, leading to the identification of a homoplasmic variant (m.10350C>A, p.Leu98Met) in *MT-ND3* gene.

Therapy with Idebenone was started 4 months after the onset at high dosage (945mg /die) and follow up showed a poor recovery in both eyes (From count finger (LE) and
0.6 (RE) to 0.025 (LE) and 0.025 (RE).

1. **Supplementary figures and tables :**

**2.1 Supplementary table 1:** pathogenicity prediction of the identified variants using online prediction software. On the right side we reported pathogenicity predictions of the main LHON primary mutations using the same tools.

| **Pathogenicity predictors:** | ***m.3632C>T*** | ***m.10350C>A*** | ***m.13340T>C*** | ***m.13379A>G*** | ***m.14538A>G*** | **Notes** | ***m.3460G>A*** | ***m.11778G>A*** | ***m.14484T>C*** |
| --- | --- | --- | --- | --- | --- | --- | --- | --- | --- |
| **Polyphen2** | Probably damaging, 0.92 | Probably damaging, 1 | Probably damaging, 1 | Probably damaging, 1 | Benign, 0 | [min 0, max 1] Neutral: score <= 0.15 Possibly damaging: 0.15 < score <= 0.85 Probably damaging: score > 0.85 | PD, 1 | PD, 1 | PD, 0.99 |
| **Provean** | Deleterious, -5.55 | Neutral, -1.92 | Deleterious, -7.21 | Deleterious, -7.21 | Deleterious, -4,3 | [min -14, max 14] Neutral: score > -2.5 Deleterious: score <= -2.5  (soft threshold) | Neutral, -2.36 | Del, -4.74 | Neutral, -1.97 |
| **MitoClass1** | Damaging | Damaging | Damaging | Damaging | Neutral | Categorical only | Damaging | Damaging | Neutral |
| **Panther** | Neutral, 0.29 | Neutral, 0.48 | Disease, 0.85 | Disease, 0.69 | Disease, 0.55 | [min 0, max 1] Neutral: score < 0.5 Disease: score >= 0.5 | Neutral, 0.26 | Disease, 0.63 | Neutral, 0.23 |
| **PhD-SNP** | Disease, 0.92 | Disease, 0.90 | Disease, 0.85 | Disease, 0.89 | Neutral, 0.49 | [min 0, max 1] Neutral: score < 0.5 Disease: score >= 0.5 | Disease, 0.72 | Disease, 0.92 | Disease, 0.92 |
| **Mtoolbox** | Deleterious, 0.73 | Deleterious, 0.71 | Deleterious, 0.82 | Deleterious, 0.87 | Neutral, 0.18 | [min 0, max 1] Neutral: score < 0.43 Deleterious: score >= 0.43 | Delet, 0.76 | Delet, 0.85 | Delet, 0.8 |

## 2.2 Supplementary Figures:


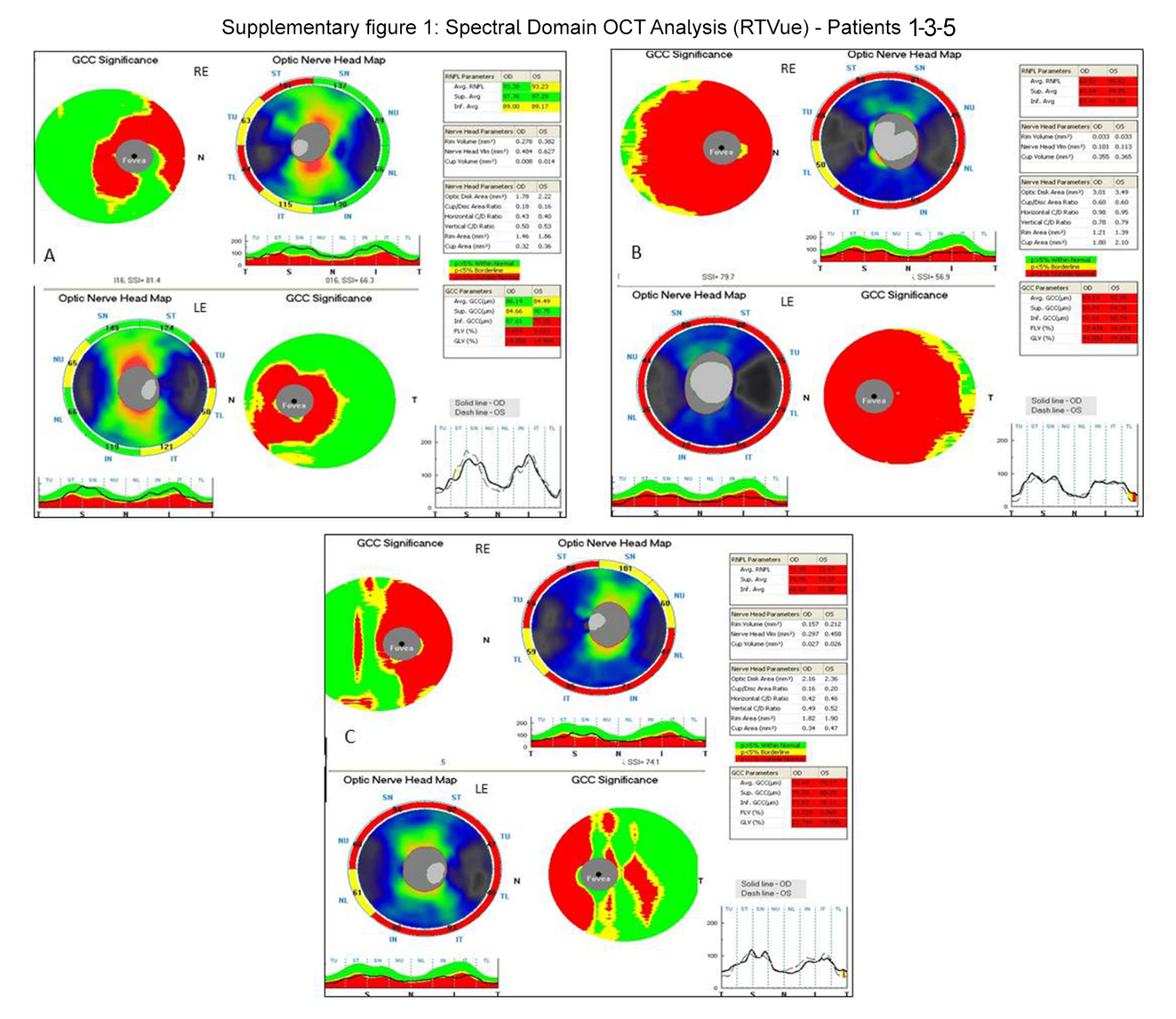


**Supplementary figure 1:** Spectral Domain OCT Analysis (RTVue) SD_OCT ANALYSIS: evolution of retinal fiber layer thickness (RNFL) and impairment of retinal ganglion cell layer (RGC). A: Patient 1; B: Patient 3; C: Patient 5.


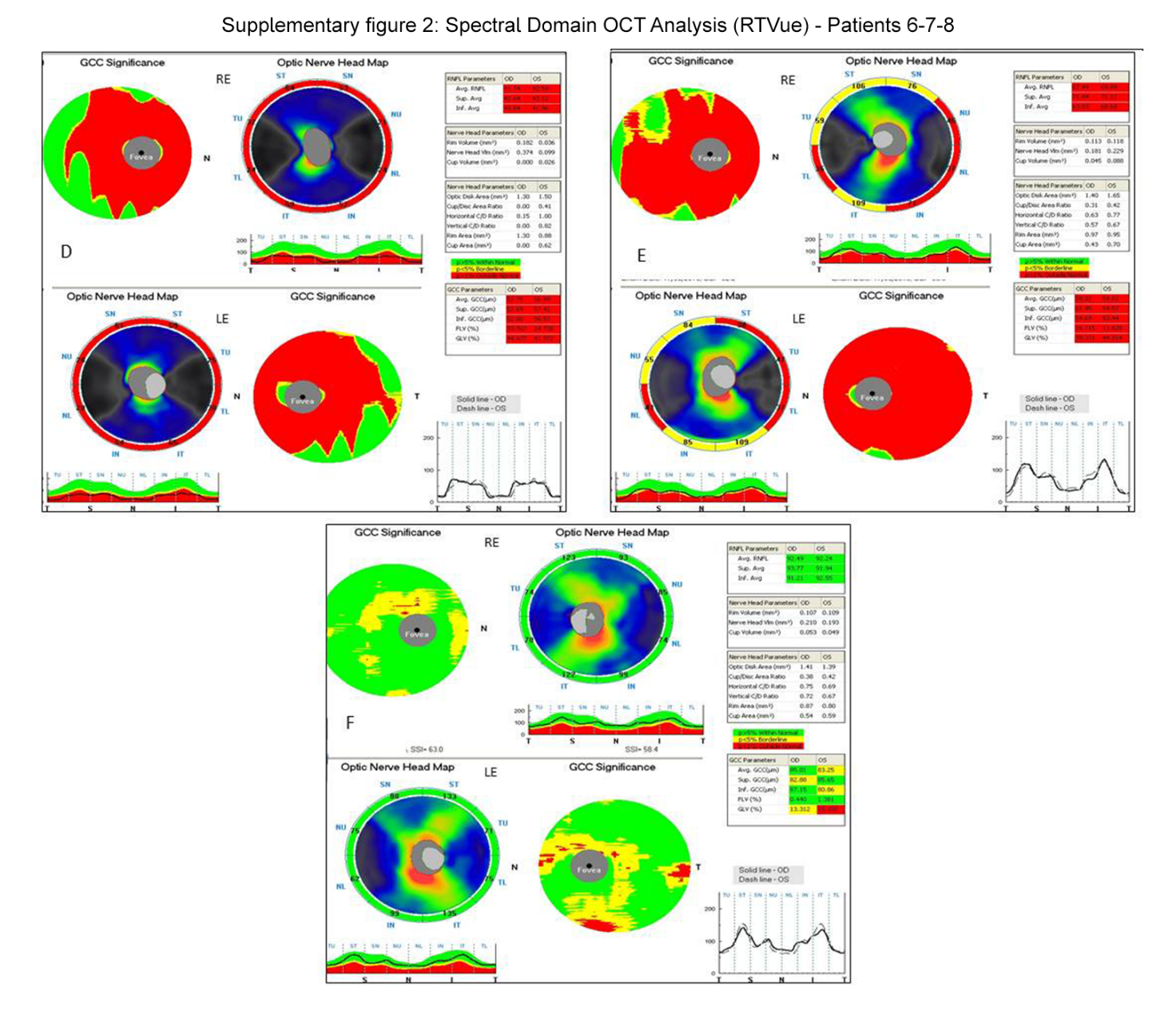
**Supplementary figure 2:** Spectral Domain OCT Analysis (RTVue) SD_OCT ANALYSIS: evolution of retinal fiber layer thickness (RNFL) and impairment of retinal ganglion cell layer (RGC). D: Patient 6; E: Patient 7; F: Patient 8.


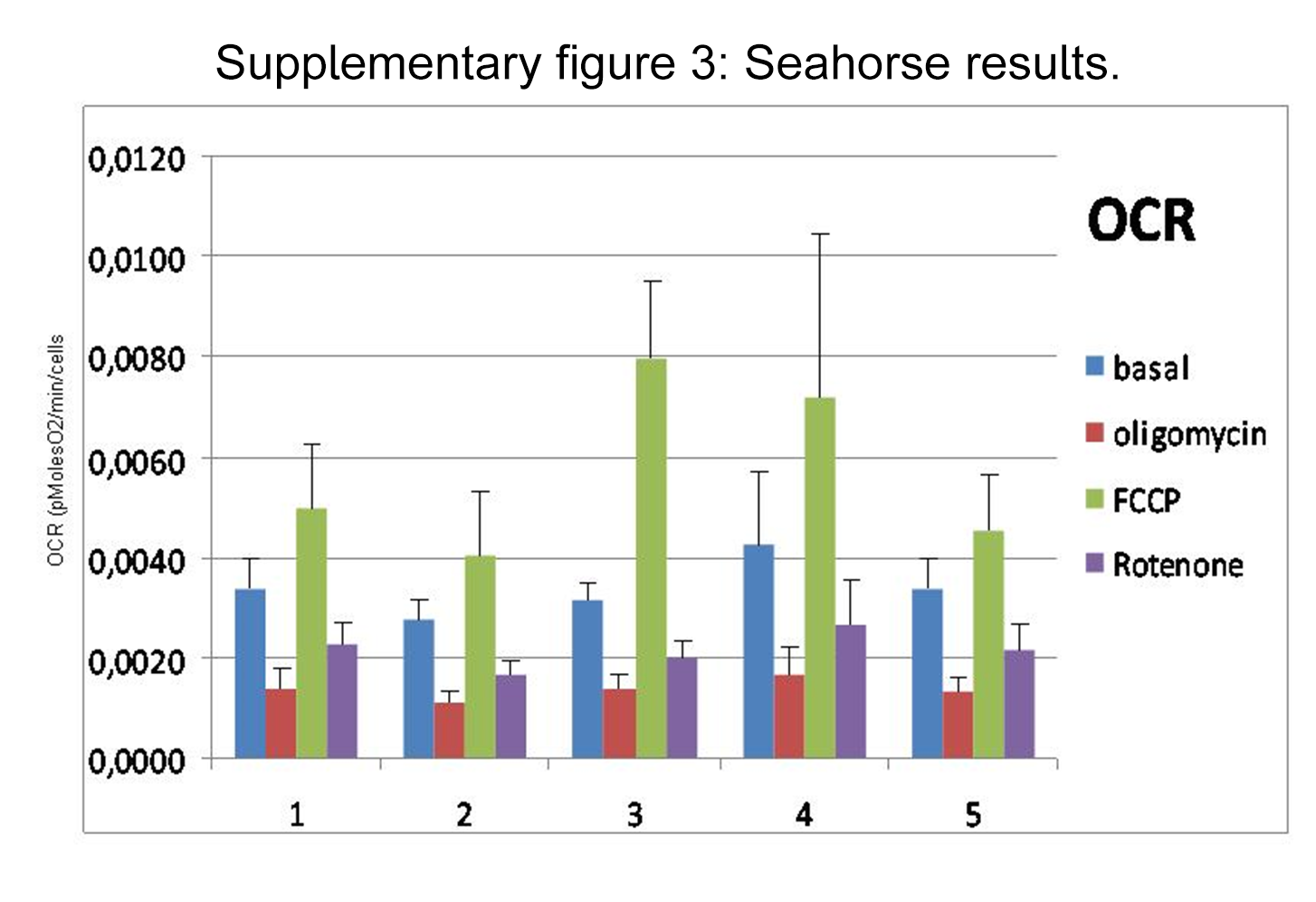
**Supplementary figure 3:** Seahorse results. OCR normalization to cell number (fibroblasts in DMEM medium). Blue, red, green and violet histograms indicate OCR-Basal, -Oligomycin, -FCCP and Rotenone, respectively. 1=Fb ctr 1; 2=Fb ctrl 2; 3=Fb Patient 3; 4=Fb Patient 5; 5=Fb Patient 6.
